# Supplementary material for: The genome sequence of flixweed, Descurainia sophia (L.) Webb ex Prantl (Brassicales: Brassicaceae)
Source: Wellcome Open Res. 2026 Sep 17;11:689. [Version 1] doi: 10.12688/wellcomeopenres.27832.1 (PMC13662381; doi:10.12688/wellcomeopenres.27832.1)
Supplement: Peer review history

# Reviewer report: Reviewer response for version 1

**DOI:** 10.21956/wellcomeopenres.30627.r169594

**Published:** 2026-10-13

**Authors:** Osamah Alisawi

**Affiliations:** 1 University of Kufa, Kufa, Iraq

High Assembly Quality and Contiguity: The Haplotype 1 assembly achieved a chromosome-level designation with 99.49% of the sequence scaffolded into 14 chromosomal pseudomolecules. It boasts a high scaffold N50 of 17.97 Mb.

 Exceptional Accuracy Metrics: The assembly significantly exceeds Earth BioGenome Project benchmarks, showing a consensus quality (QV) score of 68.6 for Haplotype 1 and a total k-mer completeness of 99.38% when combined.

 High Gene Completeness: BUSCO analysis recovered 99.5% of expected genes using the *brassicales_odb10* reference set, indicating an extremely thorough representation of the plant's gene space.

 Haplotype Resolution: The study successfully generated a phased assembly separating two distinct haplotypes using Hi-C data, which is crucial for polyploid plant species.

 Open Science Framework: The publication provides extensive transparency, fully listing all software versions, pipelines, and public repository accession numbers for open reuse.

**Unequal Haplotype Quality:** While Haplotype 1 was resolved to the chromosome level, **Haplotype 2 was only assembled to the scaffold level** and was not fully arranged into finished chromosomes.

Are the datasets clearly presented in a usable and accessible format, and the assembly and annotation available in an appropriate subject-specific repository?

Yes

Are sufficient details of the sequencing and extraction, software used, and materials provided to allow replication by others?

Yes

Are the rationale for sequencing the genome and the species significance clearly described?

Yes

Are the protocols appropriate and is the work technically sound?

Yes

Reviewer Expertise:

Plant Virology, Cytogenetic and Bioinformatics

I confirm that I have read this submission and believe that I have an appropriate level of expertise to confirm that it is of an acceptable scientific standard.

# Reviewer report: Reviewer response for version 1

**Published:** 2026-09-17

**Authors:** Automated Benchmarking

**Affiliations:** 1 Wellcome Sanger Institute, Hinxton, UK

As this article is a Genome Note, Automated Benchmarking has been used to determine whether the reported assembly passes four defined checks established by the Earth BioGenome Project: the raw and assembled data are publicly available, the Earth BioGenome Project three-part metric is met, the k-mer completeness exceeds 90%, and the BUSCO score passes the stated threshold. For Descurainia sophia (ddDesSoph1), all four checks passed. Data available: pass. ENA study PRJEB81628, raw-read accessions ERR13900456, ERR13907234, and ERR13907235, and assembly accessions GCA_965275845.1 and GCA_965275865.1 are public. Earth BioGenome Project three-part metric: pass. Haplotype 1 is reported as 6.C.Q68, meeting the 6.C.Q40 reference standard. k-mer completeness: pass. The combined haplotype assemblies have 99.38% k-mer completeness, exceeding the Earth BioGenome Project target of 90%. BUSCO: pass. Haplotype 1, which is proposed as the reference assembly for this species, has 99.5% complete BUSCOs (5.4% single-copy and 94.1% duplicated). BUSCO software version: 5.7.1; lineage dataset: brassicales_odb10. It meets the plant-specific target of greater than 90% complete BUSCOs. This decision uses values reported in the Genome Note and public accession records.

Reviewer Expertise:

NA

I confirm that I have read this submission and believe that I have an appropriate level of expertise to confirm that it is of an acceptable scientific standard.
